# Immunohistochemistry analysis of PSMA expression at prostatic biopsy in high-risk prostate cancer: potential implications for PSMA-PET patient selection

**DOI:** 10.3389/fonc.2024.1324631

**Published:** 2024-05-14

**Authors:** Matteo Droghetti, Lorenzo Bianchi, Massimiliano Presutti, Luigia Vetrone, Andrea Farolfi, Riccardo Mei, Francesca Giunchi, Alessio Degiovanni, Angelo Mottaran, Pietro Piazza, Danilo Cangemi, Paolo Castellucci, Antonietta D’Errico, Riccardo Schiavina, Eugenio Brunocilla, Stefano Fanti

**Affiliations:** ^1^ Division of Urology, IRCCS Azienda Ospedaliero-Universitaria di Bologna, Bologna, Italy; ^2^ Università degli Studi di Bologna, Bologna, Italy; ^3^ Nuclear Medicine, Alma Mater Studiorum-University of Bologna, Bologna, Italy; ^4^ Nuclear Medicine, IRCCS Azienda Ospedaliero-Universitaria di Bologna, Bologna, Italy; ^5^ Pathology, IRCCS Azienda Ospedaliero-Universitaria di Bologna, Bologna, Italy

**Keywords:** PSMA PET, immunohistochemistry, prostate cancer, biopsy, radical prostatectomy

## Abstract

**Introduction:**

Prostate-specific membrane antigen (PSMA) is a transmembrane protein expressed by normal prostatic tissue. Therefore, molecular imaging targeting PSMA (PSMA-PET) has gained particular interest and diffusion for PCa staging and restaging. Several factors may affect PSMA-PET results, and many tools have been proposed to improve patient selection. Furthermore, PSMA expression is not homogeneous among different tissues and within the prostate itself. The aims of this study were to evaluate immunohistochemistry (IHC) features of prostate biopsy samples and to assess their correlation with whole-mount specimens and PSMA-PET parameters.

**Methods:**

We included consecutive high-risk PCa patients who underwent PSMA-PET for staging proposal at our institution from January 2022 to December 2022. The PET parameters selected were SUVmax, total volume (TV), and total lesion activity (TL). Each patient underwent multiparametric MRI (mpMRI) and fusion-targeted prostate biopsy prior to surgery. IHC analyses were performed on the index lesion cores. IHC visual score (VS) (1, 2, 3) and visual pattern (VP) (membranous, cytoplasmic, and combined) and the percentage of PSMA-negative tumor areas (PSMA%neg) within biopsy cores were evaluated.

**Results:**

Forty-three patients who underwent robotic radical prostatectomy after PSMA-PET were available for analyses. Concordance between VS and VP at biopsy and final pathology showed a Cohen’s kappa coefficient of 0.39 and 0.38, respectively. Patients with PSMA%neg <20% had a higher concordance in VS and VP (Cohen’s kappa 0.49 and 0.4, respectively). No difference emerged in terms of median PSMA-TV (*p* = 0.3) and PSMA-TL (*p* = 0.9) according to VS at biopsy, while median SUVmax was higher in patients with VS 3 (*p* = 0.04). Higher SUVmax was associated with membranous and combined VP expression (*p* = 0.008). No difference emerged between patients with PSMA%neg <20% or PSMA%neg >20% on biopsy cores in terms of SUVmax, PSMA-TL, and PSMA-TV (*p* = 0.5, *p* = 0.5, and *p* = 0.9 respectively).

**Conclusions:**

We found a correlation between IHC VS and VP on targeted biopsy cores and SUVmax at PSMA-PET. However, the correlation between the IHC parameters of biopsy cores and final pathology was not as high as expected. Nevertheless, the presence of PSMA%neg <20% seems to have a better concordance in terms of visual score.

## Introduction

Prostate-specific membrane antigen (PSMA) is a transmembrane protein expressed by normal prostatic tissue. However, PSMA may be overexpressed by prostate cancer (PCa) cells. As a consequence, molecular imaging methods targeting PSMA, namely, PSMA-positron emission tomography (PET), have gained particular interest and diffusion for PCa staging and restaging, especially in high-risk patients (1, 2). PSMA-PET has led to a change in the management of PCa patients, due to significantly higher sensitivity and specificity compared with conventional imaging and other molecular imaging techniques (3–5). Indeed, the proPSMA trial showed a major change in clinical management in approximately 28% of patients with high-risk PCa in comparison to conventional imaging (1). However, the advantages of higher diagnostic accuracy, with potential “stage migration” toward oligometastatic or metastatic disease at first presentation, have yet to demonstrate a real benefit in clinical practice, particularly regarding oncological and survival outcomes. Current EAU guidelines lack outcome data of treatment changes when PSMA-PET is used to increase the sensitivity of conventional imaging (6). Several factors may affect PSMA-PET results, and many tools have been proposed to improve patient selection in this regard to maximize the benefits of this imaging modality (7–9). In fact, PSMA expression is not homogeneous among different tissues (10), and it can vary from primary PCa to distant metastases; an intratumoral heterogeneity has been in fact demonstrated, with different gradients of PSMA expression both in intra- and interpatients (11). Given these premises, growing interest arose regarding the evaluation of PSMA immunohistochemistry (IHC) features of PCa and its potential impact on PSMA-PET results. Recent studies showed the relationship between PSMA IHC expression and PSMA-PET parameters, suggesting a more accurate selection of patients with potentially higher benefit from PSMA imaging due to higher PSMA expression on pathology (10, 12, 13). However, such studies have considered IHC on the final pathological specimen after radical prostatectomy. To date, the correlation between IHC features of PSMA expression on biopsy cores and pathological specimens, with given implications on PSMA-PET results, has not been addressed yet. Therefore, we aimed to compare the PSMA expression on both prostate biopsy cores and pathological specimens with PSMA-PET findings in staging high-risk PCa patients eligible for radical prostatectomy (RP).

## Materials and methods

### Study population

In this single tertiary center retrospective study, we included 66 consecutive high-risk PCa patients who underwent prostate biopsy and ^68^Ga-PSMA-11 PET/CT (PSMA-PET) for staging proposal at our institution from January 2022 to December 2022. Overall, 15 (22.7%) patients, scheduled for external beam radiotherapy, were excluded from the analyses. Eight patients (12.1%) were excluded due to *de-novo* metastatic disease at PSMA-PET. Overall, 43 (65.2%) patients underwent robotic radical prostatectomy (RARP) with extended lymph node dissection at our institution and were included in the analysis. This study protocol was approved by the local ethics committee (244/2016/O/Oss), and each patient gave informed written consent for the use of their data.

### Prostate biopsy

Biopsies were performed in an outpatient setting with a transrectal ultrasound-guided approach, as previously described, with systematic biopsies (SBs) plus MRI-targeted biopsy (TB) (14–17). Multiparametric magnetic resonance imaging (mpMRI) was performed before biopsy in all cases. mpMRI scoring was assessed by experienced radiologists using PI-RADS v2.1 score (18). Targeted plus systematic biopsy was performed in each patient, as previously described (14, 15). SBs consisted of a typical 12-core double-sextant template from lateral to medial of the base, mid, and apex. TBs were performed for PI-RADS ≥3 lesions that were sampled with three targeted biopsy cores, using a rigid fusion platform (Aplio 500TM, Toshiba, Japan) (14, 15). All patients received oral antibiotic prophylaxis before the procedure. Periprostatic nerve blockade with lidocaine 2% was performed immediately prior to biopsy for each patient. Experienced urologists performed all the procedures.

### PET/CT imaging and analysis

Patients underwent clinical routine PET/CT after a single injection of ^68^Ga-PSMA-11 (mean dose 2 MBq/kg), according to the EANM procedure guidelines (19). Each scan was reviewed by an expert nuclear medicine physician blinded to any clinical information. The image analysis was based on the visual identification of areas with significant PSMA uptake, defined as a clear uptake above the pelvic background applying standardized criteria (20).

PSMA-PET features, i.e., PSMA total volume (PSMA-TV) expressed in cubic centimeters, and total lesion activity (PSMA-PSMA-TL), as the product of PSMA-TV and mean standardized uptake value (SUVmean) of the primary tumor, were semiautomatically extracted and collected as semiquantitative parameters, used to provide quantitative imaging biomarkers to assess the tumor burden.

SUVmean was calculated as the average of all counts in the region of interest supposed to be representative of the analyzed site. It was semiautomatically derived after drawing a spherical volume of interest (VOI) on the lesion’s area, defined as the site which ^68^Ga-PSMA uptake was significantly higher above the background.

The maximum standardized uptake value (SUVmax), PSMA-TV, and PSMA-TL were collected with an SUVmax threshold of 40% within the lesion. An SUVmax <5 was considered a negative PET (13).

### Pathology and immunohistochemistry

Prostate biopsies and RARP specimens were fixed in formalin and embedded in paraffin. From paraffin blocks, 3-μm-thick sections were cut and the slides were then stained with hematoxylin and eosin (H&E). Only the TB-positive cores and the dominant tumor lesion of the RP specimen were further analyzed with immunohistochemistry (IHC) tests. PSMA IHC was conducted with an automatic immunohistochemistry stainer instrument, as previously described (12) (Benchmark Ultra; Ventana/Roche Group 1910 Innovation Park Dr. Tucson, AZ 85755, USA). The antigen retrieval used was cell conditioning 1 for 16 min at 99°C and the primary antibody PSMA (clone EP192, prediluted, Roche) was incubated for 16 min at 36°C. The revelation system used was OptiView DAB (12 min linker and 12 min HRP multimer) (Ventana/Roche).

Histologic evaluation and PCa staging and grading were performed by a single dedicated genitourinary pathologist in accordance with the criteria established by the World Health Organization (WHO Classification of Tumours of the Urinary System and Male Genital Organs 2022) (21).

The PSMA IHC expression was evaluated both for the intensity and the site of expression, whereas this could have been granular/cytoplasmic or membranous expression or both.

### Variable definitions and outcomes

The available data consisted of the following:

- Preoperative data: age at surgery, PSA, mpMRI results, biopsy ISUP grade group, PSMA-PET results
- Pathological data: ISUP grade group, pathological T and N stage, and surgical margin status
- PSMA-PET primary tumor features: SUVmean, SUVmax, PSMA-TV, and PSMA-TL

**Figure 1:**
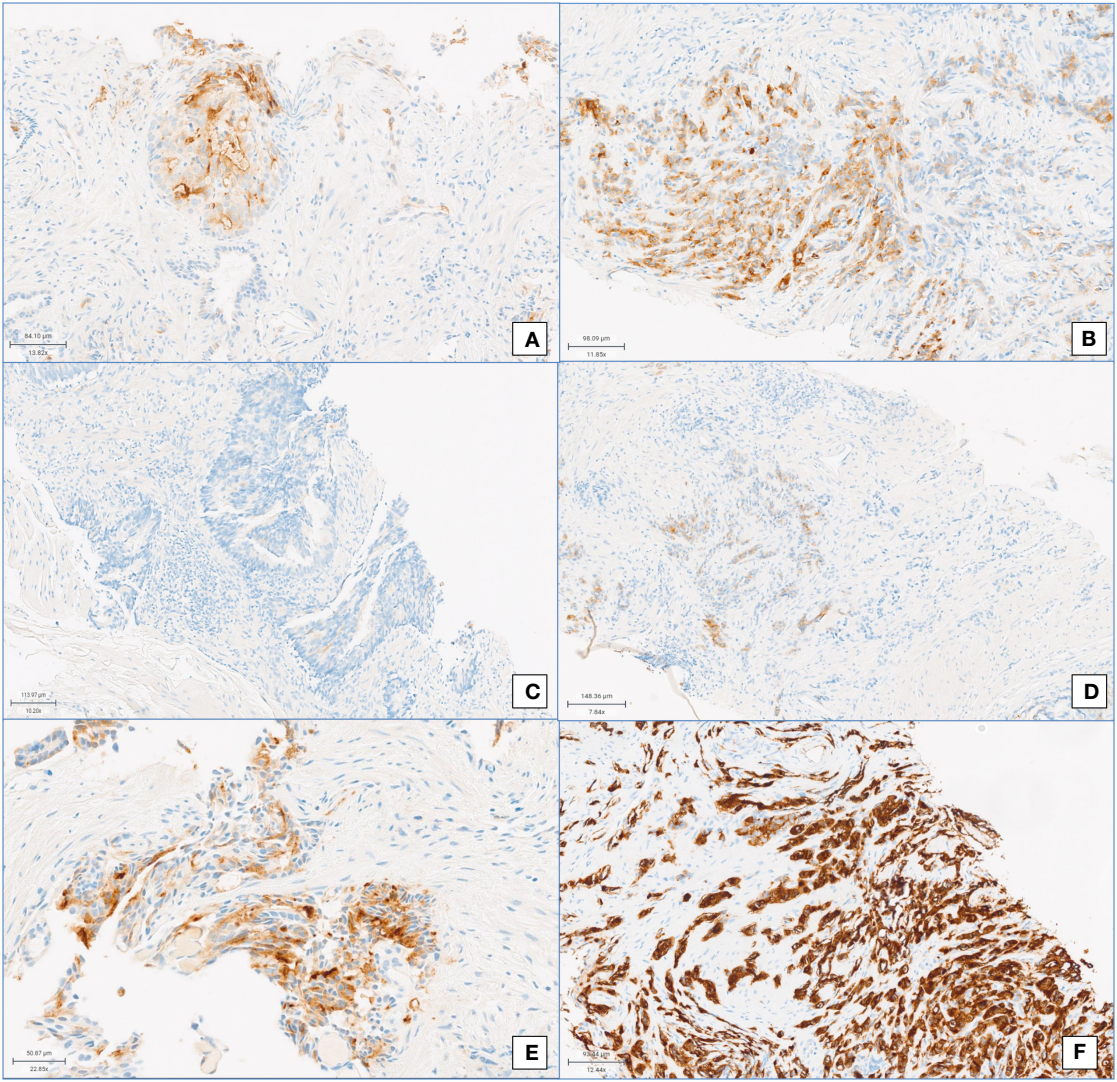
PSMA immunohistochemistry. Visual score for **(A)** membranous positivity and **(B)** cytoplasmic immunoreaction. Visual score for PSMA positivity: **(C)** score 0, **(D)** score 1+, **(E)** score 2+, and **(F)** score 3+ (both cytoplasmic and membranous positivity).

For both biopsies and the final specimen after RP, PSMA immunohistochemistry features were defined as follows:

- Visual score: The intensity of the stain for PSMA expression was visually quantified through a four-tiered system, according to the intensity of the stain compared with normal prostatic tissue, as follows: 0 = negative, 1+ = weak, 2+ = moderate, and 3+ = strong (**Figure 1**).
- Visual pattern: Immunohistochemical PSMA staining patterns, namely, membranous or cytoplasmic or combined membranous and cytoplasmic according to the site of PSMA expression.

The presence of PSMA-negative tumor areas (PSMA%neg) within the biopsy cores was further evaluated and then correlated to other IHC features and PSMA-PET features. As suggested by Rüschoff et al. (13), we utilized a cutoff value of 20%.

The primary aim of this study was to assess the concordance between the IHC features of the index lesion target biopsy cores and the primary tumor at the whole-mount final pathological examination.

The secondary aim of the study was to evaluate the correlation between IHC features of index lesion target biopsy cores and PSMA-PET parameters.

### Statistical analysis

Continuous variables were summarized using medians and interquartile range (IQR). Categorical variables were described with frequencies and proportion. Cohen’s kappa coefficient was used to assess the correlation and the concordance between the IHC features of biopsy and final pathology (visual score and visual pattern). Cohen’s kappa interpretation was reported by McHugh et al. (22) The Mann–Whitney *U*-test was used to assess the correlation between median values of PSMA-PET parameters (SUVmax, PSMA-TV, PSMA-TL) and IHC features (visual score and pattern) of prostate biopsy. Data were analyzed using SPSS Statistics (IBM, Armonk, NY, USA, v.26). An alpha value of 5% was set to be the threshold to determine statistical significance.

## Results

### Study population

**Table 1** shows the preoperative patients’ characteristics. Median age at surgery was 66 (62–72) years and the median iPSA was 9.4 ng/ml (5.9–13). At mpMRI, 69.8% of the patients had a non-organ confined (cT3) disease. Thirteen (30.2%) patients had positive pelvic lymph nodes (miN+) at PSMA-PET. At biopsy and final pathology, 37.2% and 32.6% of the patients had ISUP 5 PCa, respectively. Overall, 74.4% of the patients had locally advanced disease (pT3–4), while 30% had pN1 disease at final pathology (**Table 2**).

**Table 1:** Descriptive statistics in the overall population (*n* = 43).

**Age, years (median, IQR)**	66 (62–72)
**Preoperative PSA, ng/ml (median, IQR)**	9.4 (5.9–13)
**Biopsy ISUP grade group, *n* (%)**	
3	1 (2.3)
4	26 (60.5)
5	16 (37.2)
**Clinical stage, *n* (%)**	
cT1	11 (25.5)
cT2	27 (62.8)
cT3a	2 (4.7)
cT3b	3 (7)
**mpMRI, *n* (%)**	
Organ confined	13 (30.2)
ECE	16 (37.2)
SVI	14 (32.6)
**mpMRI PIRADS, *n* (%)**	
3	7 (18.6)
4	24 (55.8)
5	12 (27.9)
**Size lesion mpMRI, mm (median, IQR)**	12 (7–17)
Median (IQR)	
**PSMA-PET findings for T stage, *n* (%)**	
Negative	2 (4.7)
Positive	41 (95.3)
**Nodal status at PSMA-PET, *n* (%)**	
cN0	30 (69.8)
cN1	13 (30.2)
**SUVmax within the prostate**	
Median (IQR)	15 (8.5–21.7)
**PSMA-TV within the prostate**	
Median (IQR)	4 (2.7–10.8)
**PSMA-TL within the prostate**	
Median (IQR)	34.5 (14–50)

IQR, interquartile range; ISUP, International Society of Urological Pathology; PSMA, prostate-specific membrane antigen; PET, positron emission tomography; mpMRI, multiparametric magnetic resonance imaging; ECE, extracapsular extension; SVI, seminal vesicle involvement; SUV, standardized uptake value; TV, total volume; TL, total lesion; IHC, immunohistochemistry.

**Table 2:** Histologic findings and immunohistochemistry characteristics of biopsy cores and final pathologic specimens.

**IHC visual score on prostate biopsy, *n* (%)**	
0	0 (0)
1	5 (11.6)
2	10 (23.3)
3	28 (65.1)
**IHC visual pattern on prostate biopsy, *n* (%)**	
Cytosolic alone	11 (25.6)
Membranous alone	13 (30.2)
Membranous + cytosolic	19 (44.2)
**Any membranous visual pattern biopsy, *n* (%)**	
Yes	32 (74.4)
**PSMA%neg of the biopsy cores, *n* (%)**	
>20%	9 (20.9)
<20%	34 (79.1)
**IHC visual score of the pathologic specimen, *n* (%)**	
0	0 (0)
1	5 (11.6)
2	5 (11.6)
3	33 (76.8)
**IHC visual score concordance between biopsy and pathologic specimen, *n* (%)**	
No	12 (27.9)
Yes	31 (72.1)
**IHC visual score upgrade of the pathologic specimen, *n* (%)**	9 (20.9)
**IHC visual score downgrade of the pathologic specimen, *n* (%)**	3 (7)
**IHC visual pattern of the pathologic specimen, *n* (%)**	
Cytosolic alone	10 (23.3)
Membranous alone	12 (27.9)
Membranous + cytosolic	21 (48.8)
**IHC visual pattern concordance between biopsy and pathologic specimen, *n* (%)**	
No	17 (39.5)
Yes	26 (60.5)
**pISUP grade group, *n* (%)**	
2	1 (2.3)
3	11 (25.6)
4	17 (39.5)
5	14 (32.6)
**Pathologic stage, *n* (%)**	
pT2	11 (25.6)
pT3a	13 (30.2)
pT3b	17 (39.5)
pT4	2 (4.7)
**Pathologic nodal status, *n* (%)**	
pN0	30 (69.8)
pN1	13 (30.2)
**PSM, *n* (%)**	
No	24 (55.8)
Yes	19 (44.2)

IHC, immunohistochemistry; PSMA, prostate-specific membrane antigen; ISUP, International Society of Urological Pathology; PSM, positive surgical margin.

### Immunohistochemistry analysis

The results of visual scores and patterns of both biopsy and final pathologic specimens are shown in **Table 2**. In biopsy cores, 5 (11.6%), 10 (23.3%), and 28 (65.1%) had visual scores of 1, 2, and 3, respectively; in the final pathology, 5 (11.6%), 5 (11.6%), and 33 (76.8%) had visual scores of 1, 2, and 3, respectively. The concordance analysis between visual score at biopsy and final pathology showed a Cohen’s kappa coefficient of 0.393 (**Table 3A**).

**Table 3A:** Visual score of PSMA expression at IHC analysis in biopsy cores and pathologic specimens.

		T visual score (n, %)		
		1	2	3
**Biopsy visual score (n, %)**	**1**	**3 (60)**	**2 (40)**	**0 (0)**
	**2**	**1 (10)**	**2 (20)**	**7 (70)**
	**3**	**1 (3.6)**	**1 (3.6)**	**26 (92.9)**
Cohen’s kappa coefficient: 0,393				

Between biopsy and final pathology specimens, 31 (72.1%) patients had the same IHC visual score, while nine (20.9%) cases showed an IHC visual score upgrade and 3 (7%) had an IHC visual score downgrade. In biopsy cores, 11 (25.6%), 13 (30.2%), and 19 (44.2%) had cytoplasmic, membranous, and combined IHC visual patterns of PSMA expression, respectively; at the final pathology, 10 (23.3%), 12 (27.9%), and 21 (48.8%) had cytoplasmic, membranous, and combined IHC visual patterns of PSMA expression, respectively. The concordance analysis between the visual pattern at biopsy and final pathology showed minimal agreement with Cohen’s kappa (*k* = 0.383) (**Table 3B**).

**Table 3B:** Visual score of PSMA expression at IHC analysis in biopsy cores and pathologic specimens.

		T visual pattern (n, %)		
		Cytosolic	Membranous	Cytosolic + membranous
**Biopsy visual pattern (n, %)**	**Cytosolic**	**4 (36.4)**	**1 (9.1)**	**6 (54.5)**
	**Membranous**	**2 (15.4)**	**9 (69.2)**	**2 (15.4)**
	**Cytosolic + membranous**	**4 (21.1)**	**2 (10.5)**	**13 (68.4)**
Cohen’s kappa coefficient: 0,383				

Overall, 34 (79.1%) patients had PSMA%neg <20%, while 9 (20.9%) had PSMA%neg >20% in biopsy cores. In patients with PSMA%neg <20%, the correlation between biopsy and final pathology visual score showed a Cohen’s kappa coefficient of 0.49. The concordance for visual pattern in biopsy and pathological specimens was low in patients with PSMA%neg >20% (*k* = 0.12) and higher in patients with PSMA%neg <20% (*k* = 0.41) **Tables 4A**, **B**, **5A**, **B**.

**Table 4A:** Visual score correlation between biopsy cores and the final specimen in patients with PSMA%neg >20%.

		T visual score, *n* (%)		
		1	2	3
**Biopsy visual score, *n* (%)**	**1**	**1 (100)**	**0**	**0**
	**2**	**1 (20)**	**0**	**4 (80)**
	**3**	**0**	**0**	**3 (100)**

Cohen’s kappa coefficient: 0.22

**Table 4B:** Visual score correlation between biopsy cores and the final specimen in patients with PSMA%neg <20%.

		T visual score, *n* (%)		
		1	2	3
**Biopsy visual score, *n* (%)**	**1**	**2 (50)**	**2 (50)**	**0**
	**2**	**0**	**2 (40)**	**3 (60)**
	**3**	**1 (4)**	**1 (4)**	**23 (92)**

Cohen’s kappa coefficient: 0.49

**Table 5A:** Visual pattern correlation between biopsy cores and the final specimen in patients with PSMA%neg >20%.

		T visual pattern, *n* (%)		
		Cytosolic	Membranous	Cytosolic + membranous
**Biopsy visual pattern, *n* (%)**	**Cytosolic**	**1 (25)**	**1 (25)**	**2 (50)**
	**Membranous**	**1 (25)**	**3 (75)**	**0**
	**Cytosolic + membranous**	**1 (100)**	**0**	**0**

Cohen’s kappa coefficient: 0.12

**Table 5B:** Visual pattern correlation between biopsy cores and the final specimen in patients with PSMA%neg <20%.

		T visual pattern, *n* (%)		
		Cytosolic	Membranous	Cytosolic + membranous
**Biopsy visual pattern, *n* (%)**	**Cytosolic**	**3 (42.9)**	**0**	**4 (57.1)**
	**Membranous**	**1 (11.1)**	**6 (66.7)**	**2 (22.2)**
	**Cytosolic + membranous**	**3 (16.7)**	**2 (11.1)**	**13 (72.2)**

Cohen’s kappa coefficient: 0.41

### Correlation of immunohistochemistry with PET parameters

Two patients (4.7%) had a negative PSMA-PET scan, with pathological ISUP 4 and 5 and PSMA visual scores of 1 and 3, respectively. The median SUVmax in the prostate was 15 (IQR 8.5–21.7), the median PSMA-TV was 4 (IQR 2.7–10.8), and the median PSMA-TL was 34.5 (IQR 14–50).

**Figure 2:**
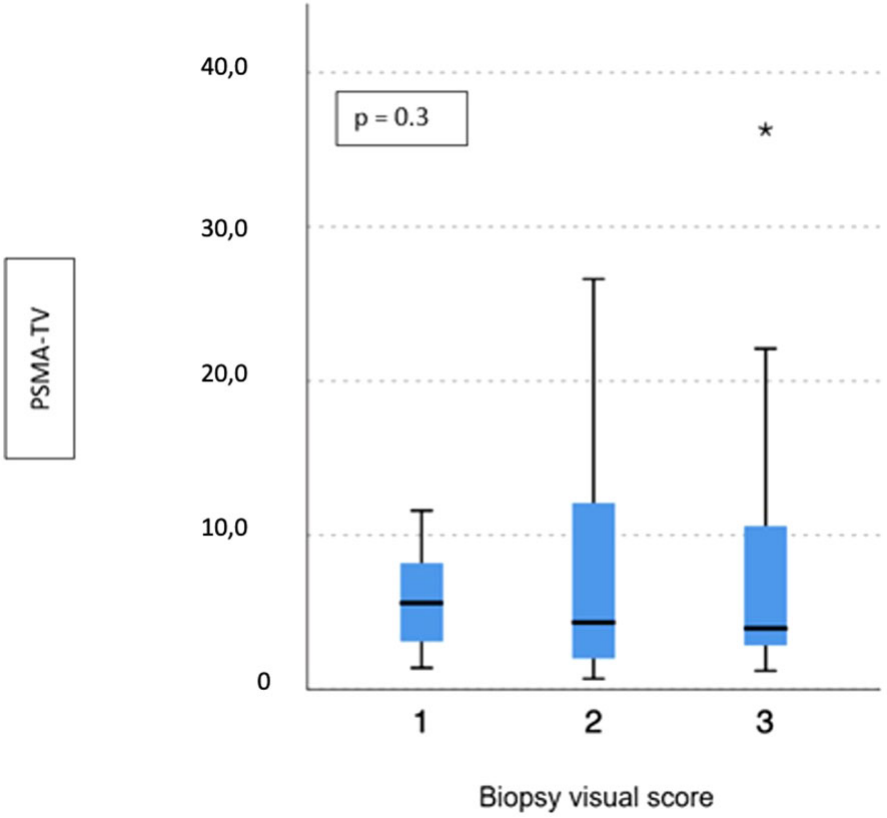
Correlation between biopsy visual score and median PSMA-TV. * means extreme values reported by sass graphics.

**Figure 3:**
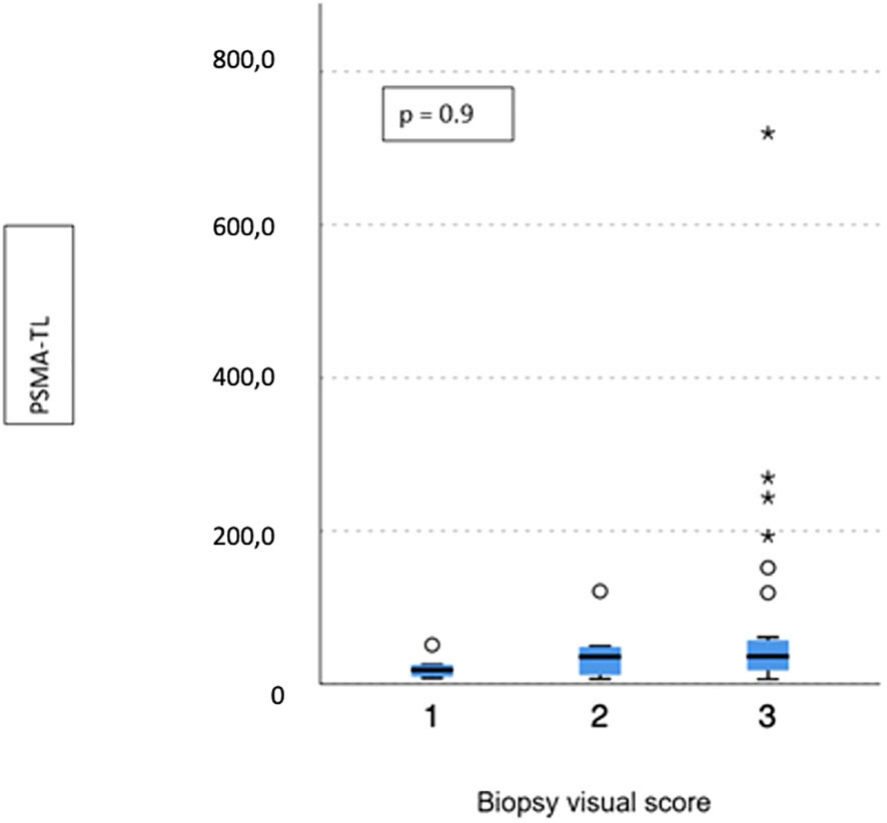
Correlation between biopsy visual score and median PSMA-TL. * means extreme values reported by sass graphics.

**Figure 4:**
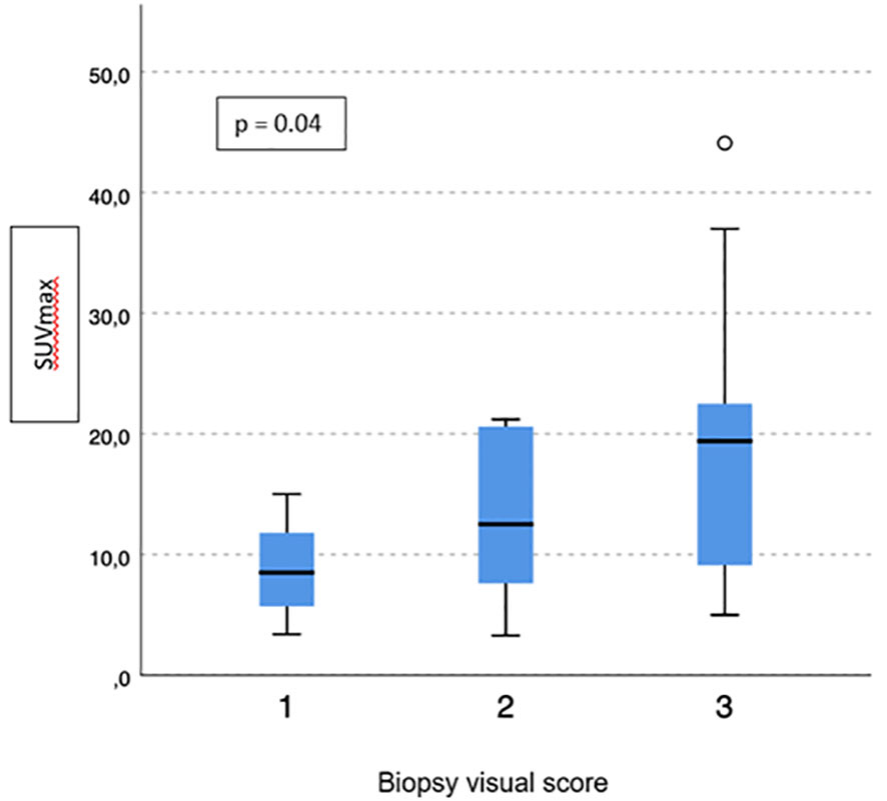
Correlation between biopsy visual score and median SUVmax.

No difference emerged in terms of PSMA-TV (*p* = 0.3) (**Figure 2**) and PSMA-TL (*p* = 0.9) (**Figure 3**) according to IHC visual score at prostatic biopsy, while SUVmax was concordant with the visual score at biopsy (*p* = 0.04) (**Figure 4**). In this regard, the pairwise comparison showed a statistically significant difference in median SUVmax between patients with a visual score of 1 and a visual score of 3 at biopsy (8.5 vs. 19.4; *p* = 0.026), while those with a visual score of 2 and 3 were found to have similar values (12.5 vs. 19.4; *p* = 0.4) as well as VS 1 and VS 2 (8.5 vs. 12.5; *p* = 1.0).

**Figure 5:**
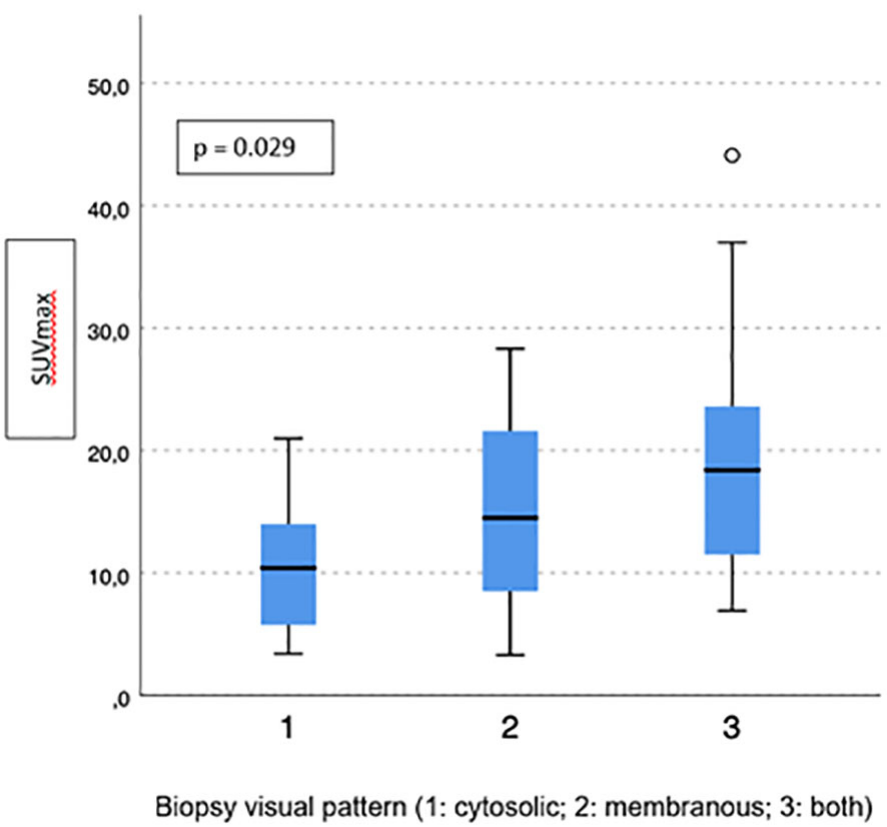
Correlation between biopsy visual pattern and median SUVmax.

**Figure 6:**
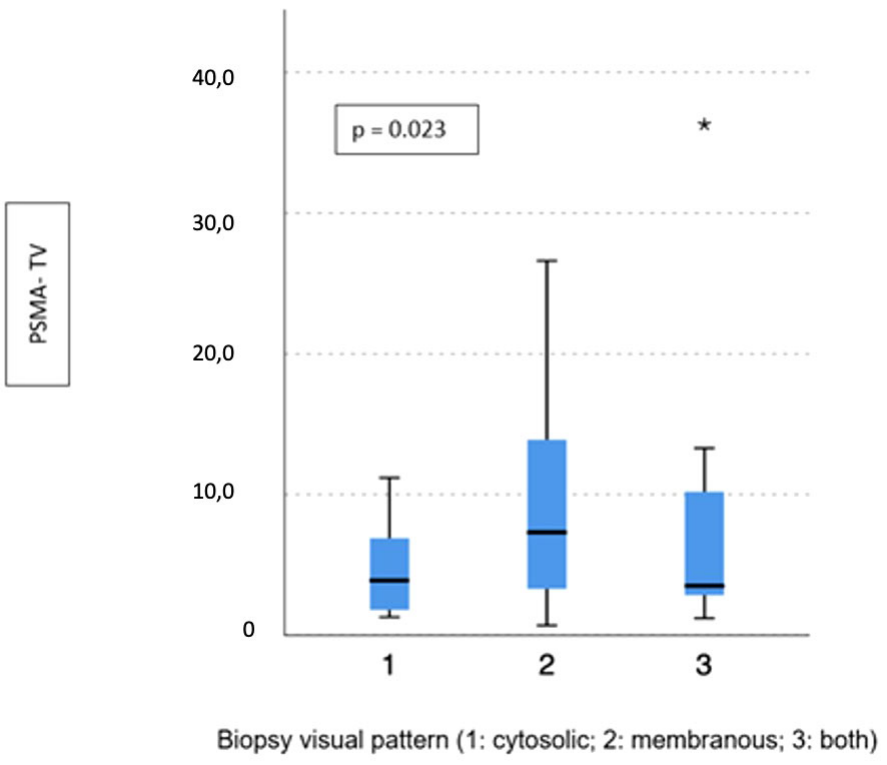
Correlation between biopsy visual pattern and median PSMA-TV. * means extreme values reported by sass graphics.

**Figure 7:**
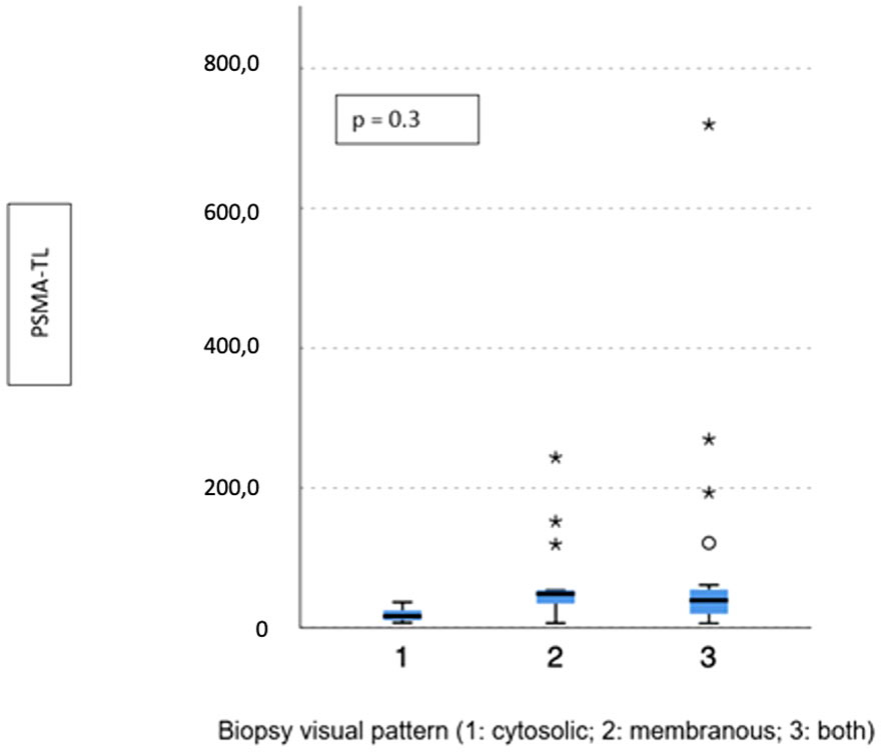
Correlation between biopsy visual pattern and median PSMA-TL. * means extreme values reported by sass graphics.

Visual pattern PSMA expression at biopsy was associated with both SUVmax and PSMA-TV at PET-PSMA (*p* = 0.029 and *p* = 0.023, respectively) (**Figures 5**, **6**), but not with PSMA-TL (**Figure 7**). In particular, higher SUVmax was found when membranous expression alone and a combined expression were present in biopsy cores (*p* = 0.008).

The presence of any membranous expression at biopsy (alone or combined with cytoplasmic) was associated with a significant difference in terms of median SUVmax (18.2 vs. 10.4; *p* = 0.02) and PSMA-TL (44.1 vs. 16.4; *p* = 0.005), but not PSMA-TV (4.4 vs. 3.9; *p* = 0.3).

No difference emerged between patients with PSMA%neg <20% or PSMA%neg >20% in biopsy cores in terms of SUVmax, PSMA-TL, and PSMA-TV (*p* = 0.5, *p* = 0.5, and *p* = 0.9, respectively).

## Discussion

PSMA-PET represents a game-changing procedure for PCa management in restaging patients with BCR after radical treatment, to target the most appropriate treatment according to disease stage and patient characteristics. Similarly, PSMA-PET may represent a prognostic tool and could replace conventional imaging in selected high-risk PCa patients before primary treatment, due to its higher diagnostic accuracy for N and M staging (1, 23); thus, increasing interest emerged for a PSMA-based treatment. Moreover, the routine adoption of PSMA-PET for each high-risk patient for staging proposal may not be available in each center and may increase health system costs; thus, a correct patient selection for PSMA-based imaging is crucial for resource optimization. In this regard, IHC examination for PSMA expression of prostatic index lesions and biopsy cores might provide important data to help physicians identify patients who might benefit the most from a PSMA-PET and avoid false-negative findings, considering that approximately 10%–15% of patients may have no PSMA expression (13).

The impact of IHC expression for PSMA on PET results is still poorly assessed, and only a few studies (10, 13, 24) have assessed the correlation between IHC features of pathologic specimens of radical prostatectomy and PSMA-PET parameters. Woythal et al. (10) showed a significantly lower SUVmax in PCa patients receiving RP with an immunoreactive score (IRS) smaller than 2 or a PSMA staining in less than 50% of the cancer cells in pathological specimens.

Rüschoff et al. (13) found a significantly lower SUVmax in patients with significant PSMA-negative tumor areas: a PSMA%neg >20% strongly correlated with a negative PSMA-PET scan and was an independent predictor for a negative PSMA-PET. However, they found no significant association between visual pattern and SUVmax.

In a recent work from our group, Vetrone et al. (12) found a correlation between high PSMA-total lesion values and PSMA expression on IHC in pathologic specimens, both for visual score (mainly for VS 1 vs. VS 2 and for VS 1 vs. VS 3) and visual pattern (membranous expression). Similarly, a higher SUVmax was associated with VS 3 and a membranous pattern.

Our results support our previous findings on IHC evaluation of pathological specimens, extending the IHC analysis also for the biopsy core of the index lesion with the purpose of selecting patients for PSMA-PET evaluation before any treatment. VS 3 on biopsy cores was associated with higher SUVmax on PET scans, potentially helping in selecting patients for PSMA-PET. Overall, biopsy core VS was poorly related to final pathology, thus limiting the use of IHC in the preoperative setting to select patients for PSMA-PET. However, for patients with <20% PSMA-negative area in the index lesion biopsy cores, the VS concordance between biopsy and the final specimen was discrete. This finding might be due to a suboptimal characterization of PCa IHC expression through biopsy cores in the case of poorly expressed PSMA PCas.

Moreover, the correlation analysis between PSMA%neg and PET parameters showed no statistically significant results, thus limiting patient selection in this context.

A good indicator for patient selection might also be represented by the PSMA expression pattern, with particular regard to membranous expression, either alone or alongside the cytoplasmic pattern. Contrary to the findings of Rüschoff et al., membranous PSMA expression pattern in biopsy cores was associated with higher SUVmax and PSMA-TL at PSMA-PET. This might be due to a higher availability of membrane receptors to the radioligand.

This discrepancy might be due to different inclusion criteria among different studies. In our series, we included only patients affected by high-risk prostate cancer, in contrast to Rüschoff et al. These patients have a more aggressive disease that is usually associated with a higher likelihood of a positive PSMA-PET scan. It is known that a Gleason score >7 is associated with a higher SUVmax at PSMA-PET scan, as described by Uprimny et al. (25) These patients are those that are usually scheduled for systemic assessment of disease burden, and it is within this cohort that insights on optimal patient selection are needed to optimize resources. However, also the PSMA expression pattern (membranous vs. cytoplasmic vs. combined) on biopsy cores was poorly related to the final pathological specimen expression patterns, making the use of IHC less reliable for patient selection.

To our knowledge, this is the first study evaluating IHC parameters analyzed on index lesions in biopsy cores and their correlation with IHC parameters at final pathology and PSMA-PET features. However, we found that IHC features of PCa in biopsy cores poorly reflect the features of the final pathological specimen. This may be due to the variability of PSMA IHC expression in PCa since PSMA IHC analysis was not performed in all bioptic specimens (but only in targeted biopsy cores) and even targeted prostatic biopsy may not be representative of the real tumor volume expression at RP, particularly in low visual score (16). Thus, it might represent a significant limitation in its usefulness for patient selection in the primary staging setting.

Moreover, our cohort included only patients with high-risk PCa, which have a low rate of negative PSMA-PET for primary disease, and the impact of IHC analyses is therefore questionable in this context to identify false-negative findings at PSMA-PET.

Future studies might extend the use of IHC analyses to determine the usefulness of the PSMA-PET scan in the biochemical recurrence (BCR) setting to detect sites of recurrence, thus identifying patients with high PSMA expression as the best candidates for PSMA imaging and PSMA-targeted treatments.

Given these noteworthy findings, our study is not devoid of limitations.

First, the number of patients included in our cohort is relatively small, and it might scarcely represent the variability of tumor PSMA expression. However, our sample was similar to those previously published on the same topic.

Second, only a few patients had a negative PSMA-PET scan for primary disease. However, all included patients were harboring a high-risk disease, thus reducing the likelihood of a negative scan.

Third, the heterogeneity of the inclusion criteria in other studies hinders a comparison with our results.

Fourth, it is possible that the retrospective nature of this study may have introduced selection bias.

Furthermore, the visual score for IHC analysis may be affected by the interoperator variability, and an objective evaluation of IHC expression by using a dedicated scanner could add to this bias.

## Conclusions

Although the correlation between IHC parameters of biopsy cores and final pathology was not as high as expected, we found a good correlation between IHC visual score and visual pattern (membranous alone or combined with cytoplasmic) of the index lesion in the targeted biopsy cores and SUVmax at PSMA-PET. Furthermore, we found that the presence of PSMA%neg <20% seems to have a better concordance between biopsy and the final specimen in terms of visual score, suggesting a possible stronger correlation between this factor and PET parameters, possibly leading to a better patient selection in this regard.

## Data availability statement

The raw data supporting the conclusions of this article will be made available by the authors, without undue reservation.

## Ethics statement

The studies involving humans were approved by IRCCS Azienda Ospedaliero-Unversitaria Policlinico S. Orsola di Bologna 244/2016/O/Oss. The studies were conducted in accordance with the local legislation and institutional requirements. The participants provided their written informed consent to participate in this study.

## Author contributions

MD: Conceptualization, Formal analysis, Methodology, Software, Writing – original draft, Writing – review & editing. LB: Conceptualization, Methodology, Supervision, Writing – original draft. MP: Data curation, Investigation, Writing – original draft. LV: Writing – review & editing. AF: Writing – review & editing. RM: Writing – review & editing. FG: Writing – review & editing. ADe: Writing – review & editing. AM: Writing – review & editing. PP: Writing – review & editing. DC: Writing – review & editing. PC: Writing – review & editing. AD’E: Writing – review & editing. RS: Writing – review & editing. EB: Writing – review & editing. SF: Writing – review & editing.

## References

[1] Hofman MS Lawrentschuk N Francis RJ Tang C Vela I Thomas P. Prostate-specific membrane antigen PET-CT in patients with high-risk prostate cancer before curative-intent surgery or radiotherapy (proPSMA): a prospective, randomised, multicentre study. Lancet Lond Engl. (2020) 395:1208–16. doi: 10.1016/S0140-6736(20)30314-7 32209449

[2] Bianchi L Schiavina R Borghesi M Ceci F Angiolini A Chessa F. How does (68) Ga-prostate-specific membrane antigen positron emission tomography/computed tomography impact the management of patients with prostate cancer recurrence after surgery? Int J Urol Off J Jpn Urol Assoc. (2019) 26(8):804–11. doi: 10.1111/iju.14012 31083784

[3] Schiavina R Bianchi L Bunocilla E. PSMA PET/CT to stage high-risk prostate cancer: Is already the time to replace conventional imaging? Minerva Urol Nephrol. (2021) 73:135–6. doi: 10.23736/S2724-6051.21.04343-5 33764031

[4] van Kalmthout LWM van Melick HHE Lavalaye J Meijer RP Kooistra A de Klerk JMH. Prospective validation of gallium-68 prostate specific membrane antigen-positron emission tomography/computerized tomography for primary staging of prostate cancer. J Urol. (2020) 203:537–45. doi: 10.1097/JU.0000000000000531 31487220

[5] Pienta KJ Gorin MA Rowe SP Carroll PR Pouliot F Probst S. A phase 2/3 prospective multicenter study of the diagnostic accuracy of prostate specific membrane antigen PET/CT with 18F-DCFPyL in prostate cancer patients (OSPREY). J Urol. (2021) 206:52–61. doi: 10.1097/JU.0000000000001698 33634707 PMC8556578

[6] Cornford P Van Den Bergh RCN Briers E Van Den Broeck T Cumberbatch MG De Santis M. EAU-EANM-ESTRO-ESUR-SIOG guidelines on prostate cancer. Part II—2020 update: treatment of relapsing and metastatic prostate cancer. Eur Urol. (2021) 79:263–82. doi: 10.1016/j.eururo.2020.09.046 33039206

[7] Bianchi L Castellucci P Farolfi A Droghetti M Artigas C Leite J. Multicenter external validation of a nomogram for predicting positive prostate-specific membrane antigen/positron emission tomography scan in patients with prostate cancer recurrence. Eur Urol Oncol. (2023) 6:41–8. doi: 10.1016/j.euo.2021.12.002 34933814

[8] Bianchi L Borghesi M Schiavina R Castellucci P Ercolino A Bianchi FM. Predictive accuracy and clinical benefit of a nomogram aimed to predict 68Ga-PSMA PET/CT positivity in patients with prostate cancer recurrence and PSA &lt; 1 ng/ml external validation on a single institution database. Eur J Nucl Med Mol Imaging. (2020) 47:2100–5. doi: 10.1007/s00259-020-04696-z 32006061

[9] Rauscher I Düwel C Haller B Rischpler C Heck MM Gschwend JE. Efficacy, predictive factors, and prediction nomograms for 68Ga-labeled prostate-specific membrane antigen-ligand positron-emission tomography/computed tomography in early biochemical recurrent prostate cancer after radical prostatectomy. Eur Urol. (2018) 73:656–61. doi: 10.1016/j.eururo.2018.01.006 29358059

[10] Woythal N Arsenic R Kempkensteffen C Miller K Janssen JC Huang K. Immunohistochemical validation of PSMA expression measured by 68Ga-PSMA PET/CT in primary prostate cancer. J Nucl Med Off Publ Soc Nucl Med. (2018) 59:238–43. doi: 10.2967/jnumed.117.195172 28775203

[11] Paschalis A Sheehan B Riisnaes R Rodrigues DN Gurel B Bertan C. Prostate-specific membrane antigen heterogeneity and DNA repair defects in prostate cancer. Eur Urol. (2019) 76:469–78. doi: 10.1016/j.eururo.2019.06.030 PMC6853166 31345636

[12] Vetrone L Mei R Bianchi L Giunchi F Farolfi A Castellucci P. Histology and PSMA expression on immunohistochemistry in high-risk prostate cancer patients: comparison with 68Ga-PSMA PET/CT features in primary staging. Cancers. (2023) 15:1716. doi: 10.3390/cancers15061716 36980602 PMC10046634

[13] Rüschoff JH Ferraro DA Muehlematter UJ Laudicella R Hermanns T Rodewald AK. What’s behind 68Ga-PSMA-11 uptake in primary prostate cancer PET? Investigation of histopathological parameters and immunohistochemical PSMA expression patterns. Eur J Nucl Med Mol Imaging. (2021) 48:4042–53. doi: 10.1007/s00259-021-05501-1 PMC8484204 34386839

[14] Borghesi M Bianchi L Barbaresi U Vagnoni V Corcioni B Gaudiano C. Diagnostic performance of MRI/TRUS fusion-guided biopsies vs. systematic prostate biopsies in biopsy-naïve, previous negative biopsy patients and men undergoing active surveillance. Minerva Urol Nephrol. (2021) 73:357–66. doi: 10.23736/S2724-6051.20.03758-3 33769008

[15] Droghetti M Bianchi L Gaudiano C Corcioni B Rustici A Piazza P. Comparison of prostate cancer detection rate at targeted biopsy of hub and spoke centers mpMRI: experience matters. Minerva Urol Nephrol. (2023) 75:42–9. doi: 10.23736/S2724-6051.22.04932-1 35766364

[16] Droghetti M Bianchi L Beretta C Balestrazzi E Costa F Feruzzi A. Site-specific concordance of targeted and systematic biopsy cores at the index lesion on multiparametric magnetic resonance: can we spare the double-tap? World J Urol. (2023) 41:27–33. doi: 10.1007/s00345-022-04229-3 36471133

[17] Schiavina R Droghetti M Novara G Bianchi L Gaudiano C Panebianco V. The role of multiparametric MRI in active surveillance for low-risk prostate cancer: The ROMAS randomized controlled trial. Urol Oncol Semin Orig Investig. (2021) 39:433.e1–7. doi: 10.1016/j.urolonc.2020.10.018 33191117

[18] Turkbey B Rosenkrantz AB Haider MA Padhani AR Villeirs G Macura KJ. Prostate imaging reporting and data system version 2.1: 2019 update of prostate imaging reporting and data system version 2. Eur Urol. (2019) 76:340–51. doi: 10.1016/j.eururo.2019.02.033 30898406

[19] Fendler WP Eiber M Beheshti M Bomanji J Calais J Ceci F. PSMA PET/CT: joint EANM procedure guideline/SNMMI procedure standard for prostate cancer imaging 2. 0. Eur J Nucl Med Mol Imaging. (2023) 50:1466–86. doi: 10.1007/s00259-022-06089-w PMC10027805 36604326

[20] Seifert R Emmett L Rowe SP Herrmann K Hadaschik B Calais J. Second version of the prostate cancer molecular imaging standardized evaluation framework including response evaluation for clinical trials (PROMISE V2). Eur Urol. (2023) 83:405–12. doi: 10.1016/j.eururo.2023.02.002 36935345

[21] Netto GJ Amin MB Berney DM Compérat EM Gill AJ Hartmann A. The 2022 world health organization classification of tumors of the urinary system and male genital organs-part B: Prostate and urinary tract tumors. Eur Urol. (2022) 82:469–82. doi: 10.1016/j.eururo.2022.07.002 35965208

[22] McHugh ML. Interrater reliability: the kappa statistic. Biochem Medica. (2012) 22:276–82. doi: 10.11613/issn.1846-7482 PMC3900052 23092060

[23] Perera M Papa N Roberts M Williams M Udovicich C Vela I. Gallium-68 prostate-specific membrane antigen positron emission tomography in advanced prostate cancer—Updated diagnostic utility, sensitivity, specificity, and distribution of prostate-specific membrane antigen-avid lesions: A systematic review and meta-analysis. Eur Urol. (2019) 77(4):403–17. doi: 10.1016/j.eururo.2019.01.049 30773328

[24] Ferraro DA Rüschoff JH Muehlematter UJ Kranzbühler B Müller J Messerli M. Immunohistochemical PSMA expression patterns of primary prostate cancer tissue are associated with the detection rate of biochemical recurrence with 68Ga-PSMA-11-PET. Theranostics. (2020) 10:6082–94. doi: 10.7150/thno.44584 PMC7255040 32483440

[25] Uprimny C Kroiss AS Decristoforo C Fritz J von Guggenberg E Kendler D. 68Ga-PSMA-11 PET/CT in primary staging of prostate cancer: PSA and Gleason score predict the intensity of tracer accumulation in the primary tumour. Eur J Nucl Med Mol Imaging. (2017) 44:941–9. doi: 10.1007/s00259-017-3631-6 28138747

